# Engaging hospitalised patients in their nutrition care using technology: development of the NUTRI-TEC intervention

**DOI:** 10.1186/s12913-020-5017-x

**Published:** 2020-02-27

**Authors:** Shelley Roberts, Zane Hopper, Wendy Chaboyer, Ruben Gonzalez, Merrilyn Banks, Ben Desbrow, Andrea P. Marshall

**Affiliations:** 1 School of Allied Health Sciences, Griffith University, Gold Coast Campus, Southport, QLD 4222 Australia; 2 Gold Coast Hospital and Health Service, 1 Hospital Blvd, Southport, QLD 4215 Australia; 3 Menzies Health Institute Queensland, Griffith University, Gold Coast Campus, Southport, QLD 4222 Australia; 4 School of Nursing and Midwifery, Griffith University, Gold Coast Campus, Southport, QLD 4222 Australia; 5 School of Information and Communication Technology, Griffith University, Gold Coast Campus, Southport, QLD 4222 Australia; 6 Royal Brisbane and Women’s Hospital, Cnr Butterfield St and Bowen Bridge Road, Herston, QLD 4029 Australia

**Keywords:** Complex interventions, Health information technology, Hospital patients, Integrated knowledge translation, Nutrition, Patient participation, Patient engagement

## Abstract

**Background:**

Nutrition is vital for health and recovery during hospitalisation, however most patients fail to meet minimum dietary requirements and up to 50% of patients are malnourished in hospital. When patients participate in nutrition care, their dietary intakes are improved. Advances in health information technology (HIT) have broadened the ways by which patients can participate in care. Our team has developed an innovative, HIT-based intervention (called NUTRI-TEC; engaging patients in their nutrition care using technology), facilitating patient participation in their nutrition care in hospital. This paper aims to describe the systematic and iterative process by which the intervention was developed.

**Methods:**

NUTRI-TEC development was informed by the Medical Research Council guidance for developing complex interventions and underpinned by theoretical frameworks and concepts (i.e. integrated knowledge translation and patient participation in care), existing evidence and a rigorous program of research. The intervention was co-developed by the multidisciplinary research team and stakeholders, including health consumers (patients), health professionals and industry partners. We used an iterative development and evaluation cycle and regularly tested the intervention with hospital patients and clinicians.

**Results:**

The NUTRI-TEC intervention involves active patient participation in their nutrition care during hospitalisation. It has two components: 1) Patient education and training; and 2) Guided nutrition goal setting and patient-generated dietary intake tracking. The first component includes brief education on the importance of meeting energy/protein requirements in hospital; and training on how to use the hospital’s electronic foodservice system, accessed via bedside computer screens. The second component involves patients recording their food intake after each meal on their bedside computer and tracking their intakes relative to their goals. This is supported with brief, daily goal-setting sessions with a health care professional.

**Conclusions:**

NUTRI-TEC is a HIT intervention designed to enable patient participation in their nutrition care in hospital. As research on HIT interventions to engage patients in health care in the hospital setting is in its infancy, and as gaps and inconsistencies in the development of such interventions exist, this paper will inform future development of HIT-based interventions in the hospital setting.

## Contributions to the literature

- Health information technology (HIT) is becoming an integral component of health care delivery, however limited literature exists on the development of HIT interventions, particularly in hospitals. As HIT interventions are often multifaceted and delivered in complex settings with heterogeneous populations, more research is needed on their development.
- This paper describes how research frameworks, theory and evidence guided the iterative, co-development of a complex HIT intervention aiming to engage hospitalised patients in their nutrition care, to improve dietary intakes.
- The development process reported here will be useful to researchers and clinicians aiming to develop/implement technology-related complex interventions in the future.

## Background

Malnutrition is a major problem in hospitals, affecting 20–50% of patients worldwide [1, 2]. It results in poor patient outcomes including increased risks of infection [3], pressure injury [4], reduced mobility and falls [5, 6] and mortality [7]; and contributes to increased length of hospital stay, readmissions and costs [7, 8]. Malnutrition can be prevented or corrected with adequate dietary intake. In fact, inadequate food intake is the major modifiable risk factor for malnutrition and an independent risk factor for mortality among hospitalised patients [9, 10]. Due to a complex mix of patient and organisational factors, achieving optimal nutrition intake during hospitalisation is difficult and most patients fail to meet nutrition requirements in hospital [1, 10, 11]. For example, reduced dietary intakes due to poor appetite, personal preferences or nutrition impacting symptoms, in addition to increased metabolic requirements due to medical conditions, are all patient-related factors contributing to malnutrition [12]. Organisational factors such as the hospital foodservice, mealtime environment and the way hospitals and staff provide nutrition care (e.g. screening, assessment, intervention, monitoring, documentation, communication) also impact patients’ nutrition [12, 13]. Given that hospital malnutrition is a complex problem, multifaceted interventions are required to address it.

Patient participation in their own care results in improved health outcomes [14] and increased patient safety and satisfaction with care [15]. Patient participation is a core aspect of safe and quality health care [16], is one of the Australian Commission on Safety and Quality in Health Care’s national standards [17] and is endorsed by the World Health Organization’s Patients for Patient Safety movement [18]. Preliminary research has shown patient participation in nutrition care is a feasible and effective strategy for improving dietary intakes in hospitals [19, 20].

Advances in health information technology (HIT) are broadening the ways by which patients can participate in their health care and as a result, the safety, quality and cost-effectiveness of care is likely to be improved [21]. A systematic review of 170 studies found technology-based health interventions had a positive effect on patient engagement, health behaviours and health outcomes (such as weight loss, exercise tolerance and blood glucose control) among patients with a range of health conditions [21]. However, most studies are community-based and there have been calls to undertake similar research in the hospital setting [22]. Hence, our team has developed an innovative, technology-based intervention to engage hospitalised patients in their own nutrition care, termed NUTRI-TEC (engaging hospital patients in their nutrition care using technology).

## Methods

### Study overview

The aim of the overall program of research was to systematically develop an intervention for improving nutrition among hospitalised patients, by enabling them to participate in their care. The aim of this paper is to describe the rigorous process used to develop the intervention, adhering to the TIDieR reporting guidelines. Due to a change in the study context while the research was underway (the introduction of a new electronic foodservice system at the hospital), the intervention was developed in two major stages: 1) development of the original intervention (with paper-based materials); and 2) adaptation of the intervention to the new technology. Intervention development was guided by research frameworks and informed by both theory and evidence/data. The multidisciplinary research team engaged with key stakeholders to co-develop the intervention, which was done in an iterative development-evaluation cycle. This section outlines the methodology used in intervention development. A figure depicting the project timeline can be seen in Supplementary Materials.

### Study setting and participants

The research was conducted at a large, metropolitan tertiary teaching hospital in Queensland, Australia in collaboration with the hospital’s university partner. There were three levels/types of participants in the research: 1) study team; 2) key stakeholders; and 3) hospital patients/ staff. The ways in which these participant groups were engaged in the research are explained in subsequent sections. Ethical approval was obtained for each original study (i.e. usability testing and patient/staff interview studies), the details of which can be found in the associated publications [23, 24].

### Research frameworks and approaches

This research was informed by the UK’s Medical Research Council (MRC) guidance for developing and evaluating complex interventions [25] and the Knowledge to Action (K2A) process [26]. The MRC framework was chosen to provide methodological rigour for the development and evaluation of the intervention, as it is a new and untested innovation. The framework suggests interventions should be theory and evidence-based and undergo adequate pilot testing prior to evaluation of effectiveness.

The research also used an integrated knowledge translation (iKT) approach [27] guided by the K2A process [26]. A key feature of iKT research is co-production of knowledge through engagement of knowledge end-users (i.e. patients, staff) throughout the entire research process. This was done to ensure barriers to knowledge use were assessed within the local context and intervention strategies were relevant, appropriate and acceptable. This project engaged end-users in three ways. Firstly, we ensured we had adequate representation of end-users on the study team by including clinician researchers from the study hospital. Secondly, we engaged in regular discussions/consultations with key hospital stakeholders (representing nutrition/dietetics, nursing, foodservices, information technology) and our industry partner (software company) to feed results of each phase back and gain insights into the data and how it should be used to inform the intervention. Finally, we conducted end-user studies with patients and staff to explore usability and perceptions of using the intervention to engage patients in their nutrition care [23, 24].

### Theoretical underpinnings and evidence base

As per the MRC guidance and K2A process, the intervention was both theory- and evidence-based. The original (paper-based) intervention was informed by theories/concepts of patient participation in care and self-efficacy; and additional theories on HIT design, usability and engagement were used in the intervention’s adaptation. The theories used were supported by previous research, with literature reviews occurring at both stages of intervention development, as well as data from our previous observational and qualitative studies [28–31]. This section outlines the theories and evidence used in the intervention’s design.

#### Patient participation in care

The intervention was heavily underpinned by the concept of patient participation in care, defined by four core dimensions: 1) a good relationship between patient and health care professional (HCP); 2) surrendering of some power/control by HCPs; 3) meaningful information/knowledge exchange between patient and HCP; and 4) active mutual engagement in health care activities [32]. These concepts were supported by evidence suggesting patient participation in care results in better health outcomes, improved patient safety and higher satisfaction with care [14, 15]. While there was limited evidence on patient participation in nutrition care in hospital, one study showed improvements in dietary intakes when patients participated by recording their food intake and engaging in nutritional goal setting with nurses [19]. These strategies aligned with the core dimensions of participation outlined above and showed promise for improving dietary intakes; hence, were considered as potential strategies for the intervention.

#### Self-efficacy

Bandura’s theory on self-efficacy is based on the notion that one’s belief in their own ability to organise and execute actions required to manage situations (i.e. achieve goals) influences their thoughts, actions and emotional responses, ultimately determining whether the desired outcome is achieved or not [33]. Self-efficacy is shown to be a powerful predictor of health behaviour, including food choices and adherence to nutrition interventions [34]. One study found that self-regulatory behaviours such as setting goals, monitoring food intake and planning for nutrition-related challenges were the best predictors of participants’ nutrition [35]. Hence, authors suggested nutrition interventions should focus on improving the use of such behaviours [35]. The experience of mastery, or “enactive attainment”, is the most important factor determining self-efficacy; success raises self-efficacy, while failure lowers it [33]. This was observed in our original pilot study, where patients expressed in post-intervention interviews that the intervention increased their awareness, motivation and responsibility for improving their nutrition intake in hospital [20]. They said the increased knowledge/awareness of their nutrition intake (and how to improve it) and goals, and seeing improvements in their intake day-to-day encouraged and motivated them to keep going, and increased feelings of ownership and responsibility for their nutrition in hospital [20].

#### HIT development and evaluation

Prior to adapting the intervention to HIT, we conducted a realist review to evaluate the use of HIT to engage hospitalised patients in their care [29]. The review identified five key features of interventions successfully engaging patients in care (information sharing; self-assessment and feedback; tailored education; user-centred design; support in use of HIT) and analysed these in terms of context, mechanisms and outcomes [29]. These findings informed the design of the intervention and its evaluation, as the review found most studies did not adequately assess HIT usability. Key theories and supporting data on HIT were also reviewed in preparation for the intervention’s adaptation. For example, the Technology Acceptance Model postulates that perceived usefulness and ease of use are the main predictors of technology acceptance [36], which is supported by findings of a systematic review of HIT interventions [37]. Hence, theoretical knowledge on HIT design and evaluation (with particular focus on user-centred design/usability) informed the intervention’s adaptation [38–41]. Importantly, an iterative development and evaluation cycle [40, 41] was employed to ensure high usability (described in further detail below).

## Results

### Developing intervention components

From the theory and evidence outlined above, two intervention components (and several sub-components) were developed: 1) Patient education and training; and 2) Patient-generated food intake monitoring and nutritional goal-setting. The first component included a brief education on the importance of meeting energy and protein requirements in hospital; and training on how to use the hospital’s electronic foodservice system, accessed via bedside computer screens. The second component involved patients recording their food intake after each meal on their bedside computer and monitoring their dietary intakes relative to their nutrition goals (supported with brief, daily goal-setting sessions with a dietitian). An overview of the intervention components and how they were informed is outlined in Table 1.

**Table 1:** Intervention components and supporting theory/evidence

Intervention component	Patient participation^a^	Self- efficacy^b^	HIT evidence^c^
Component 1: Patient education and training			
Education (meeting nutrition requirements in hospital)	• Meaningful exchange of knowledge/information • Active mutual engagement in health care activities • Good relationship established between patient and HCP	• Enactive attainment (mastery experience) • Verbal persuasion/ encouragement	• Information sharing • Tailored education
Training (using bedside computer to track food intake and view/monitor goals)			• Support in use of HIT
Component 2: Patient participation in nutrition care (intake tracking and goal setting)			
Intake tracking (patient-generated food intake monitoring)	• Good relationship between patient and HCP • Meaningful exchange of knowledge/information • Surrendering of power/ control by HCPs • Active mutual engagement in health care activities	• Enactive attainment (mastery experience) • Verbal persuasion/ encouragement	• Information sharing • Self-assessment and feedback • User-centred design • Support in use of HIT
Goal setting (regular dietitian-guided nutritional goal setting)			

^a^Based on Sahlsten’s concept analysis of patient participation in care [26]

^b^Based on Bandura’s theory of self-efficacy [27]

^c^Based on realist review of inpatient HIT interventions [29]

#### Patient education and training

Education and training are foundational to many health interventions and both are core functions of behaviour change in Michie’s Behaviour Change Wheel [42]. Similarly, knowledge is the first stage of the adoption process in Rogers’ Diffusion of Innovations theory [43]. As such, education on the importance of meeting nutrition needs in hospital was a core component of the original intervention. This education was delivered to the patient face-to-face by a trained dietitian, supported with paper resources, and took around 10 mins to deliver. Patients found this useful; in follow-up interviews they highlighted the importance of the education for increasing their knowledge and understanding of nutrition for recovery [20]. For this reason, patient education (delivered by a trained dietitian upon enrolment in the study) remained a core component in the adapted NUTRI-TEC intervention. This was supported by findings from our realist review (tailored education was a key feature of HIT interventions engaging hospitalised patients in care [29]) and by Sahlsten’s concept analysis (meaningful exchange of knowledge/information is a core concept of patient participation in care [32]). In addition, in our realist review we found that supporting patients in the use of HIT was key to intervention success; hence, we included training (delivered at the same time as the education) on how to use the patient portal to enter food intake and view/monitor nutrition goals.

#### Participation in nutrition care

Assessing the dietary intakes of hospitalised patients is a challenge, with the most accurate methods being costly and time-consuming. Twenty-four hour recall is a method commonly used by dietitians to elicit nutrition intake from a patient; however, remembering what they have eaten may be difficult for patients who are confused, drowsy, overwhelmed, or who find days in hospital hard to differentiate. Nurses are often asked to keep food charts for patients, however these have poor completion and accuracy [44]. Meanwhile, most patients themselves are an underutilised resource and many are well positioned to record their own dietary intake. Patient-generated food intake tracking has dual benefits; it enables patient participation in care, which has been shown to improve patients outcomes [14] and releases staff time (e.g. nurses, dietitians and their assistants) to enable other care tasks.

The original intervention involved patients recording their food intakes on a paper food chart and engaging in guided nutritional goal setting with a trained dietitian for 3 days. Adaptation to the NUTRI-TEC intervention involved the same activities, but instead patients recorded their food intake and viewed/monitored their nutrition goals via an electronic patient portal, accessed by bedside computer screens. The portal was built into the hospital’s existing electronic foodservice system, through which patients already ordered their meals. The system contained the nutrition content of all foods offered by the hospital foodservice. In the ‘My Meal Ordering’ page (Fig. 2), patients could view the nutrition content of each menu item and select items based on this. Once they submitted their order, they could also see the total energy and protein content of their order for each meal, and for each day.

At the back end of the system, dietitians entered patients’ individually estimated energy and protein requirements, which patients could view in the ‘My Nutrition Goals’ page. After each meal, patients could select how much they consumed of each meal item (i.e. none, ¼, ½, ¾, all) in the ‘My Food Intake’ page (Fig. 3). The system automatically calculated each patient’s total nutrient intake (as entered by the patient) and presented this as a percentage of their nutrition requirements (entered by the dietitian) in the ‘My Nutrition Goals’ page (Fig. 4). This page automatically updated after patients entered their intakes for each meal, could be viewed by patients at any time, and was used by dietitians during the daily goal-setting sessions. It displayed patients’ nutrition requirements (goals) and intakes in both numerical and graphical format, as well as showing which meals had been entered so far that day, and the amount of energy (kJ) and protein (grams) needed to meet their goal.

### Developing the patient portal

Once the intervention components had been refined, they were incorporated into a patient portal, accessed via patient bedside computer screens. This process was guided by literature on HIT design, such as the System Development Life Cycle [40, 41], which involves four main stages to be completed prior to routine use of a HIT program: specification (needs assessment), component development, integration of components into a system, and integration of a system into the intended environment. At each step, testing is conducted and the components/system are iteratively refined.

#### Stage 1 – specification

Specifying needs for the setting and users occurred over a significant time period (several years) in two main stages. First, a literature review of previous research, findings from our own observational and qualitative research [28, 30, 31] and relevant theories informed development of the original paper-based intervention, which was piloted in hospital. Patients who were able to participate (cognitively intact adults who understand basic English) and most likely to benefit from the intervention (those at nutritional risk with length of stay ≥4 days) were the target population. Findings from the pilot study indicated the intervention was feasible, acceptable to patients and likely to be effective in improving nutrition intakes [20]; and economic analyses indicated it was cost-effective [45, 46]. These data were presented to relevant hospital managers who considered the intervention (and its adaptation to HIT) a reasonable investment, as it was likely to streamline clinical care and improve patient outcomes. The work was also deemed worthwhile by our funding body, as the lead author won a competitive research fellowship to conduct the intervention’s adaptation; and by our industry partner, who agreed to provide in-kind software design support. Second, adaptation of this intervention to technology was informed by: a realist review of in-hospital HIT interventions [29]; data from the original pilot study including qualitative patient feedback [20]; frequent meetings and discussions among the study team and key stakeholders including HIT experts and hospital clinicians (dietitians, foodservice managers, nurses); and regular liaison with our industry partner. Also during this stage, early ideas for the NUTRI-TEC intervention were presented to health consumers, who provided feedback.

#### Stage 2 – component development

The system component development involved developing mock-up versions of the intervention components and undertaking basic system performance and validity testing. The research team, which included dietitians (from research, clinical and foodservice backgrounds), nurses and information technology (IT) experts, worked in close collaboration with industry partner Delegate Technology (Vienna, Austria) to develop the mock-up components. Members of the research team (SR, ZH and RG) undertook basic system-task testing to further refine the components. We then undertook usability testing with patients [24], which involved patients navigating completing each task while using the ‘Think Aloud’ technique [47], which is often used in HIT usability testing [40]. This enables researchers to capture what participants are thinking during the performance of a task, which is often lost if questions are asked after task completion [47]. Usability testing was conducted with 32 hospital patients who were a direct match to intended users of the intervention (consistent eligibility criteria used across studies) and was immediately followed by a semi-structured interview exploring patients’ perceptions of using this technology to participate in their hospital nutrition care. While in-depth findings are reported elsewhere [24], in summary we found: being familiar with technology increased confidence with using NUTRI-TEC components but wasn’t essential (as most patients found it easy to use); user interface design and perceived benefits of the program impacted patients’ acceptability of it; patients thought the program could enable participation in their care; and participation in care occurred to varying extents. We also conducted interviews with hospital staff (doctors, nurses, dietitians, nutrition assistants and foodservice staff) to explore their perceptions of the mock-up NUTRI-TEC components. Findings are published elsewhere [23], but briefly, we found: staff accepted and promoted patient participation in care and thought this intervention would be a useful tool to do this; and staff strove for optimal nutrition care and thought NUTRI-TEC could improve information access/management to support patient-centred care. Staff also discussed considerations for implementing a program like this in practice.

#### Stage 3 – combination of components into a system

Patient and staff interview findings (summarised above) informed this next step, which involved combining the components into complete system. The study team worked in close collaboration with our industry partner, hospital clinicians and hospital IT staff to make many changes to the user interface and functionality of the software. This involved making dozens of detailed, individual software update requests to our industry partner, to address specific form and function (i.e. layout and functional) issues identified in Stage 3. After each request was actioned, that aspect of the software was tested with clinical dietitians from the study hospital. Over time, all components were integrated into a complete working system. This was a time and resource-intensive process, which took approximately 12 months to complete.

#### Stage 4 – integration of system into environment

**Fig. 1:**
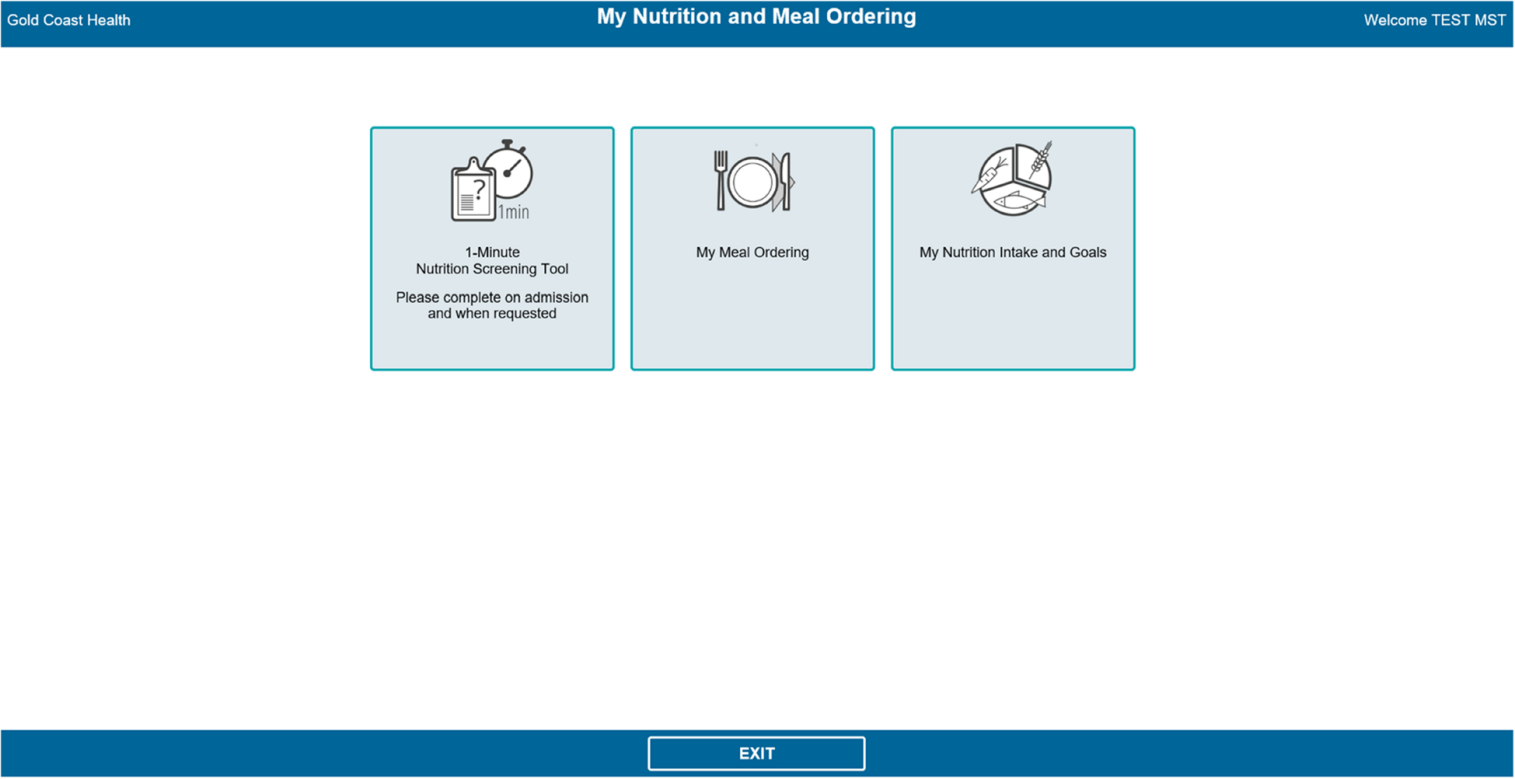
Home screen

**Fig. 2:**
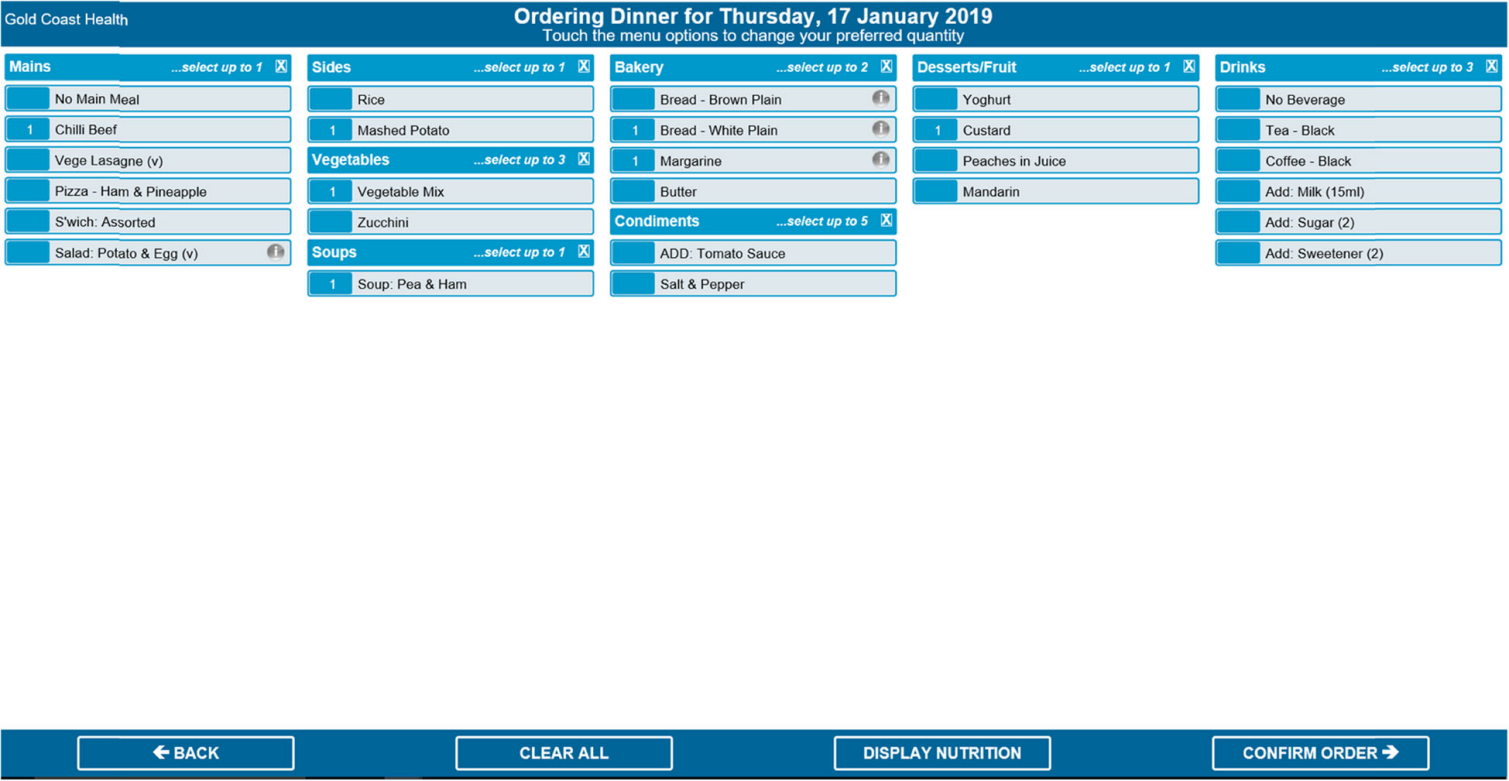
My Meal Ordering home page

**Fig. 3:**
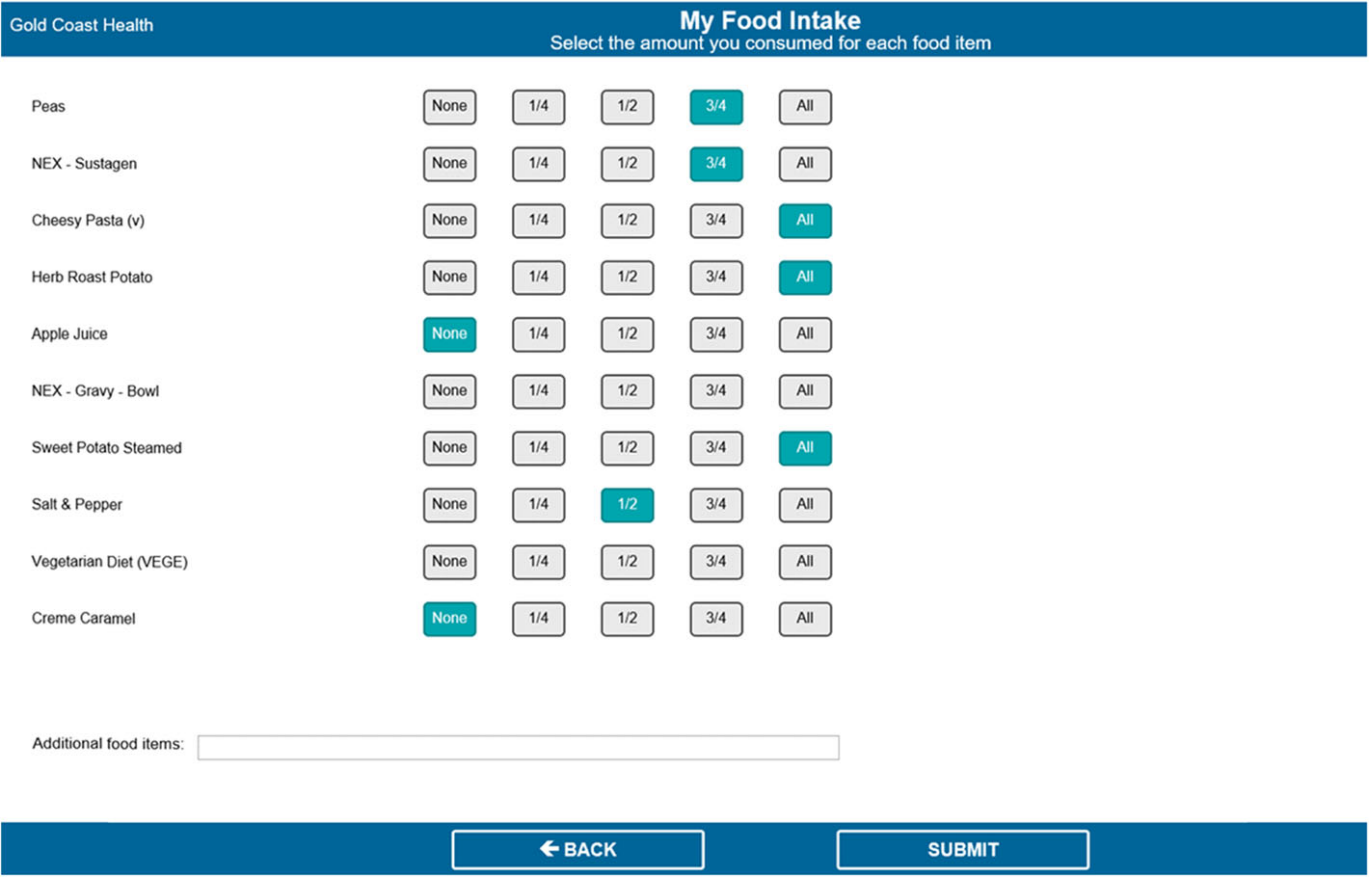
My Food Intake (intake tracking) page

**Fig. 4:**
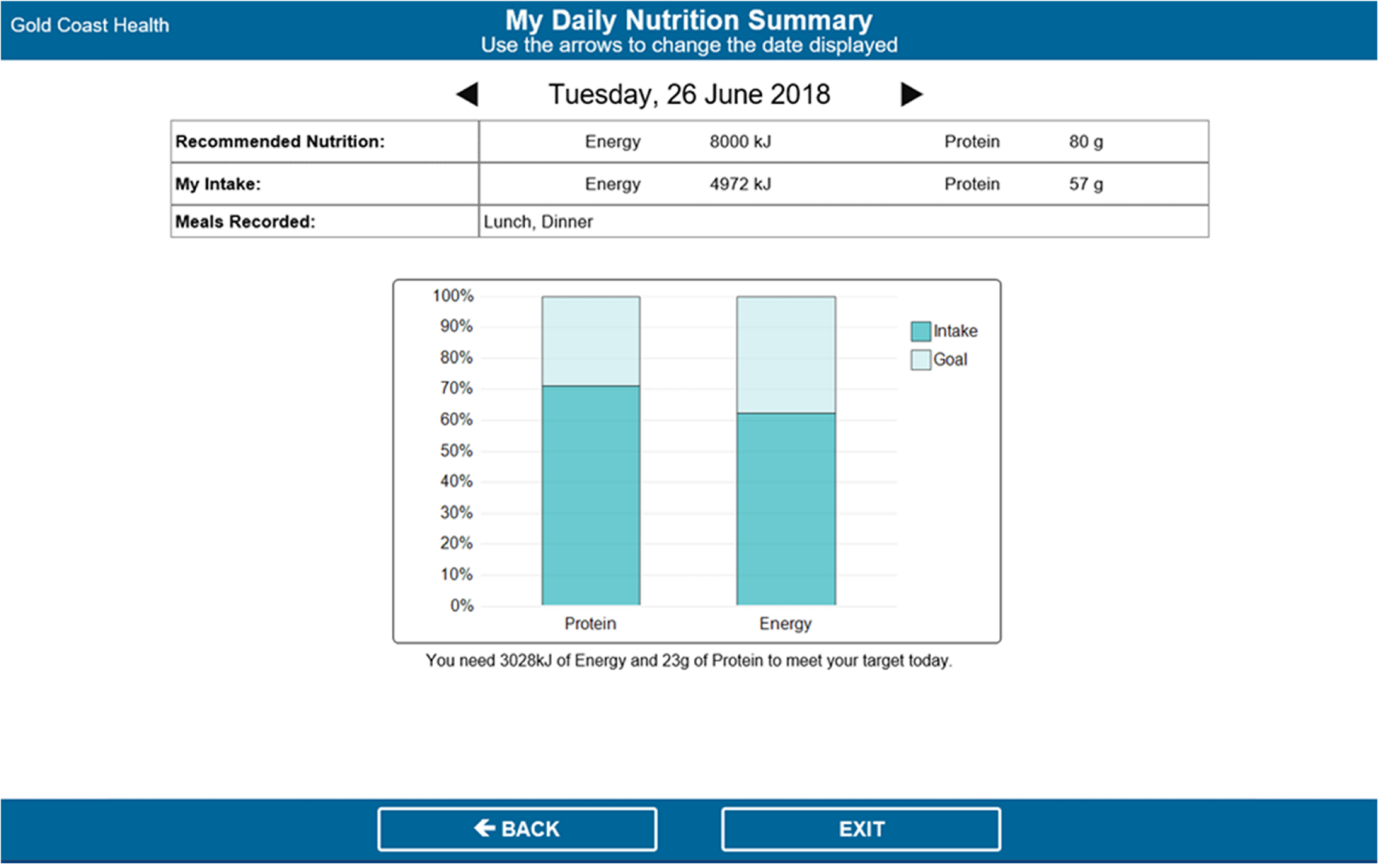
My Nutrition Goals page

Once the complete version of the software was developed by Delegate Technology, it was sent to the study hospital. The research team closely liaised with clinical and foodservice dietitians and IT staff from the study hospital to integrate the software into the hospital’s IT system, ensuring the layout and design were appropriate (i.e. text and icons were displayed clearly, no text was lost when displayed on patients’ bedside computer screens, etc.). This process took approximately 6–8 weeks. Figures 1, 2, 3, and 4 show the final patient interface of the system, which was used in a pilot (feasibility) study of the intervention as a whole. The pilot study has been completed and will be reported elsewhere. Stage 5 (routine use) will not be applicable until the intervention is ready for adoption into usual practice (i.e. after being evaluated in a trial).

Note (Fig. 2): If the ‘Display Nutrition’ button was selected, energy (kJ) and protein (grams) contents of each meal item were displayed for the patient.

## Discussion

This paper describes the iterative, co-development of NUTRI-TEC; a HIT intervention aiming to improve patients’ nutrition intakes by engaging them in nutrition care during hospitalisation. The intervention was underpinned by research frameworks, theory and evidence; had extensive end-user testing and input; and was piloted in the hospital setting with real patients. The intervention comprises aspects of patient education, patient participation/engagement in care, individualised/tailored care and HIT. It involves educating patients on the importance of meeting their nutrition requirements in hospital; and engaging them in their nutrition care by monitoring their personalised nutrition goals and self-recording their dietary intake.

Use of research frameworks ensured NUTRI-TEC was developed in a rigorous way and met end-user needs. For example, the MRC framework for developing and evaluating complex interventions [25] was used to ensure it was evidence-based, grounded in theory and underwent adequate piloting in preparation for a larger trial. The K2A cycle [26] and an iKT approach [27] were used to ensure NUTRI-TEC was suitable for the local context and relevant and acceptable to end-users such as hospital patients and staff. This iKT/co-development approach was especially important in NUTRI-TEC’s development, as the intervention targets hospital patients; a population and setting known for being complex and difficult to translate evidence into practice [48, 49]. In our realist review we identified that user-centred design was a key feature of successful HIT interventions aiming to engage hospitalised patients in care [29]. However, it is recognised in literature that many HIT interventions lack adequate input from IT experts or are not designed with HIT development theory [21]. For example, a systematic review of HIT interventions found that only 47% of studies explicitly referenced theory and only 34% conducted usability testing [21], suggesting HIT interventions are often designed in an ad-hoc way. We not only included HCPs and IT experts on our study team, but also conducted several rounds of usability testing and evaluation with end-users (patients and hospital staff), with findings incorporated into NUTRI-TEC’s design. This iterative process, guided by appropriate HIT theory and supported by input from our IT expert and industry partner, is a major strength of the NUTRI-TEC intervention and addresses several limitations of previous studies.

The use of person-centred care, patient participation/engagement and individualised/tailored care approaches is another strength of NUTRI-TEC. While definitions of person-centred care vary in the literature, common themes include access to health information, respect for patients’ individual needs and preferences, and involvement in all aspects of care including decision-making processes [16]. While NUTRI-TEC mainly focuses on patient involvement in care through participation in nutrition goal setting and dietary intake tracking, it also enables access to nutrition information; both general (i.e. importance of meeting nutrition requirements in hospital and nutrition values of all menu items) and individualised (i.e. patients’ own nutrition requirements). It also allows patients’ individual needs and preferences to be considered alongside this information (i.e. patient menu selection with full knowledge of all options), so patients can make informed decisions. These concepts align with national and international recommendations on consumer engagement and person-centred care [16, 18], with evidence suggesting improved health outcomes are associated with these [14, 15]. A systematic review found that higher patient participation in condition self-management using HIT was correlated with greater improvements in health outcomes [21]. Further, by allowing patients to do simple tasks themselves (such as dietary intake monitoring), NUTRI-TEC may result in time savings for nurses, nutrition assistants and dietitians; and streamlined care planning and delivery.

While the process used to develop the NUTRI-TEC intervention was systematic and rigorous, which is likely to increase its feasibility, acceptability and effectiveness, there are limitations to this approach. Firstly, the process was time and resource intensive, spanning 3–4 years and requiring several small grants and a dedicated team (including one full-time research fellow who spent ~ 80% of their time on the project for 2 years) to complete. This was mainly due to the inclusion of technology; as software development, user and system testing, and hospital implementation took longer than expected. As hospitals are a complex and ever-changing environment, the long timeframe over which the research was conducted meant that organisational changes had to be accounted for throughout. The research was facilitated by securing a study team with the appropriate skills and expertise, access to a university hospital where testing could take place, and having an industry partner on board who was willing to undertake most of the IT development in-kind. Others may have difficulty in securing these stakeholders and resources. While this process is not feasible to do in usual clinical practice, it is recommended that development of new HIT interventions follow a rigorous research process. That is, HIT interventions must be theory and evidence based, have input from IT experts and follow established HIT design principles, to ensure interventions are successful and resources used for their development are not wasted.

## Conclusions

This paper describes how research frameworks (MRC framework and K2A process), theory and theoretical concepts (iKT, patient engagement/participation in care) and local data can be used to develop an innovative, technology-based intervention for improving nutrition among hospitalised patients. The iterative co-development of the intervention was time and resource intensive, but is required to increase its likelihood of being relevant, appropriate and acceptable to end-users; and hence, effective and sustainable in practice. Reporting on this process and our learnings will benefit others aiming to develop complex health interventions (especially those using technology) in the future. The next phase of the research will involve testing the clinical and cost effectiveness of the NUTRI-TEC intervention for improving nutrition intake among hospitalised patients.

## Supplementary information

**Additional file 1: Figure S1.** Project timeline with System Development Life Cycle stages.

## Data Availability

Not applicable.

## Abbreviations

HCP: Health care professional HIT Health information technology iKT Integrated knowledge translation IT Information technology K2A Knowledge to Action KT Knowledge translation MRC Medical Research Council NUTRI-TECs Engaging hospitalised patients in their nutrition care using technology
