# Supplementary material for: Engaging hospitalised patients in their nutrition care using technology: development of the NUTRI-TEC intervention
Source: BMC Health Serv Res. 2020 Feb 27;20:148. doi: 10.1186/s12913-020-5017-x (PMC7045423; doi:10.1186/s12913-020-5017-x)
Supplement: Supplementary file 1 — Additional file 1: Figure S1. Project timeline with System Development Life Cycle stages. [file 12913_2020_5017_MOESM1_ESM.docx]

| **2012–2014** | **2014** | **2015** | **2016*** | **2017*** | **2018** |
| --- | --- | --- | --- | --- | --- |

| Original intervention development and piloting (PhD study) | Introduction of EFS at study hospital | Funding application | Study team development, industry partner engagement | Realist review of inpatient HIT interventions | Mock-up versions of intervention components developed | Patient usability testing and patient/staff interviews | Development of NUTRI-TEC program as a whole system | Implementation of NUTRI-TEC program into hospital system, pilot testing |
| --- | --- | --- | --- | --- | --- | --- | --- | --- |
|  | | | Specification | | Component development | | Combination of components into a system | Integration of system into environment |

Figure S1: Project timeline with System Development Life Cycle stages

*Research fellow funded 2016 and 2017, with ~80% of time spent on the NUTRI-TEC study

EFS Electronic foodservice system
